# Update on diagnostic procedures in third window syndromes

**DOI:** 10.1007/s00106-024-01467-2

**Published:** 2025-07-31

**Authors:** Julia Dlugaiczyk, Sebastian Rösch, Georgios Mantokoudis

**Affiliations:** 1 Department of Otorhinolaryngology, Head and Neck Surgery & Interdisciplinary Center of Vertigo, Balance and Ocular Motor Disorders, University Hospital Zurich (USZ), University of Zurich (UZH), Rämistrasse 100, 8091 Zurich, Switzerland; 2 Department of Otorhinolaryngology, Head and Neck Surgery, Paracelsus Medical University, Salzburg, Austria; 3 Department of Otorhinolaryngology, Head and Neck Surgery, University Hospital Regensburg, Regensburg, Germany; 4 Department for Otolaryngology, Head and Neck Surgery, Inselspital University Hospital Bern, Bern, Switzerland

**Keywords:** Audiometry, Electrocochleography, Enlarged vestibular aqueduct, Superior canal dehiscence syndrome, Vestibular evoked myogenic potentials, Audiometrie, Syndrom der oberen Bogengangsdehiszenz, Elektrocochleographie, Erweiterter vestibulärer Aquädukt, Vestibulär evozierte myogene Potenziale

## Abstract

**Background:**

The diagnosis of third window syndromes often poses a challenge in clinical practice.

**Objective:**

This paper provides an overview of diagnostic procedures in third window syndromes, with special emphasis on superior canal dehiscence syndrome (SCDS), large vestibular aqueduct syndrome (LVAS), and X-chromosomal malformation of the cochlea.

**Materials and methods:**

A literature search was performed in PubMed up to December 2023. Furthermore, a selection of the authors’ own cases is presented.

**Results:**

Audiovestibular tests for the diagnosis of third window syndromes are most often reported for patients with SCDS in the literature. In this context, cut-off values with different sensitivities and specificities have been defined for different outcome parameters of vestibular evoked myogenic potentials. Current developments include the application of electrocochleography, broadband tympanometry, video head impulse testing, and vibration-induced nystagmus. Genetic analyses are increasingly applied in LVAS.

**Conclusion:**

The diagnosis of third window syndromes is always based on the synthesis of patients’ symptoms, clinical signs, audiovestibular test results, and imaging.

## Abstract (de)

**Hintergrund:**

Die Diagnostik von Drittfenstersyndromen stellt in der klinischen Praxis häufig eine Herausforderung dar.

**Ziel der Arbeit:**

Die vorliegende Arbeit gibt einen aktuellen Überblick über diagnostische Optionen bei diesen Krankheitsbildern, mit besonderem Fokus auf das Syndrom der oberen Bogengangsdehiszenz (SCDS), das Syndrom des erweiterten vestibulären Aquädukts (LVAS) und die X‑chromosomale Malformation der Cochlea.

**Material und Methoden:**

Dazu erfolgte eine Literaturrecherche in der Datenbank PubMed bis Dezember 2023 und die Aufarbeitung eigener Fälle.

**Ergebnisse:**

Audiovestibuläre Testverfahren zur Diagnose eines Drittfenstersyndroms werden in der Literatur am häufigsten im Rahmen des SCDS beschrieben. Für vestibulär evozierte myogene Potenziale wurden hier Grenzwerte mit unterschiedlichen Sensitivitäten/Spezifitäten für verschiedene Messparameter definiert. Neuere Entwicklungen umfassen die Anwendung der Elektrocochleographie, der Breitbandtympanometrie, des Video-Kopfimpulstests und des vibrationsinduzierten Nystagmus. Beim LVAS kommen zunehmend genetische Analysen zum Einsatz.

**Schlussfolgerung:**

Die Diagnose eines Drittfenstersyndroms ergibt sich immer aus der Synthese von Symptomen, klinischen Zeichen, apparativen Untersuchungsbefunden und der Bildgebung.

Third window syndromes are characterized by additional orifices besides the oval and round window in the bony labyrinth, resulting in altered biomechanics and fluid dynamics within the inner ear. The subsequent pathognomonic audiovestibular signs and symptoms are highly variable between different disorders, and even between different patients with the same diagnosis.

## Current diagnostic procedures

The present article provides an overview of current knowledge about diagnostic procedures in third window syndromes, in particular the following entities: superior canal dehiscence syndrome (SCDS), large vestibular aqueduct syndrome (LVAS), and X‑chromosomal malformations of the cochlea. Besides commonly applied methods (e.g., pure-tone audiometry [PTA], vestibular evoked myogenic potentials [VEMPs], imaging), we will also cover investigations that are not yet routinely used in this context, such as electrocochleography (ECochG), wideband tympanometry, video head impulse testing (vHIT), vibration-induced nystagmus (VIN), and genetic analyses.

## Third window syndromes: overview

**Fig. 1:**
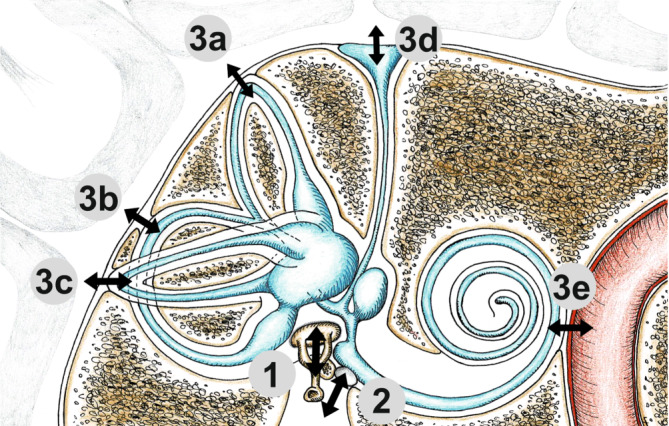
Schematic representation of possible locations of a third window in the bony labyrinth. (Courtesy © Dr. med. Hergen Friedrich, all rights reserved). In addition to the oval window (*1*) and the round window (*2*), an additional third window (*3*) may occur at the following locations, among others: superior (*3a*), posterior (*3b*), lateral (*3c*) semicircular canal, enlarged vestibular aqueduct (*3d*), or bony dehiscence between the cochlea and the carotid canal (*3e*)

A detailed overview has been presented by Dlugaiczyk [7]. In summary, a distinction can be made between third window syndromes that result from the enlargement of a natural neurovascular foramen (e.g., enlarged vestibular aqueduct [EVA]; “incomplete partition type III”) and those with a non-natural opening of the otic capsule (e.g., semicircular canal dehiscence: superior > posterior > lateral in descending frequency; bony dehiscence between the cochlea and the carotid canal, Fig. 1).

There are also diffuse lesions of the bony labyrinth that may lead to weakening of the otic capsule

While the majority of third window syndromes can be attributed to a clearly localized defect (see examples above), there are also diffuse lesions of the bony labyrinth that together may lead to a weakening of the otic capsule in the sense of a dehiscence (e.g., osteogenesis imperfecta or fibrous dysplasia of the temporal bone). A further distinction is made between congenital and acquired third window syndromes, as well as between primary and secondary forms. The latter are found in inflammatory (e.g., cholesteatoma), infectious (e.g., otosyphilis), vascular (e.g., paraganglioma), and neoplastic (e.g., plasmacytoma, Langerhans cell histiocytosis, tumors of the endolymphatic sac) destructive processes of the temporal bone.

The additional “third” window in the bony labyrinth creates a *locus minoris resistentiae*, leading to changes in the biomechanics and fluid dynamics of the cochlea and vestibular organ [6, 7, 17]. The resulting pathognomonic symptoms, clinical signs, and audiovestibular test findings are summarized in Table 1 for SCDS, and also apply to other third window syndromes. In this context, special attention should be paid to inner ear conductive hearing loss with partially negative bone conduction (BC) thresholds and a maximum air–bone gap (ABG) between 250 and 500 Hz in the pure-tone audiogram (PTA; [24]).

**Table 1:** ICVD criteria for superior canal dehiscence syndrome [40]

**Fig. 2:**
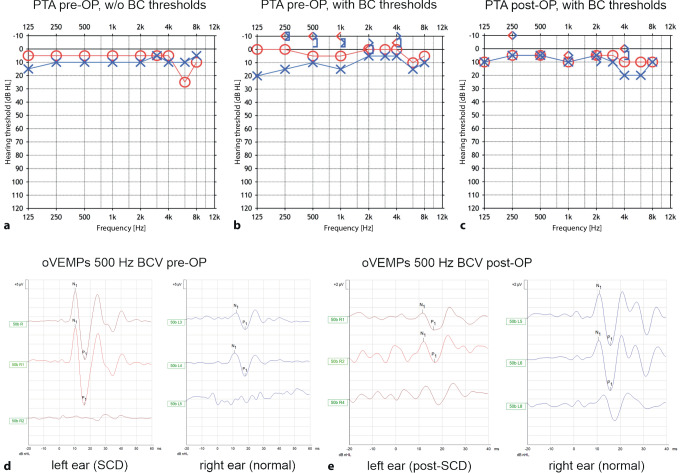
Preoperative (**a,** **b,** **d**) and postoperative (**c,** **e**) audiovestibular findings in a patient with left superior canal dehiscence syndrome (*SCDS*). The dehiscence was closed by transmastoid plugging. **a** Preoperative pure-tone audiogram (*PTA*) without bone conduction (*BC*) thresholds: normal bilateral air conduction (*AC*) thresholds. **b** Preoperative PTA with AC and BC thresholds: negative bilateral BC thresholds (see text for further explanation). **c** Postoperative PTA with normalization of BC thresholds. **d** Preoperative ocular vestibular evoked myogenic potentials (*oVEMPs*) for a 500 Hz BC stimulus at Fz (midline of the forehead at the hairline): oVEMP n10p15 amplitude (*N1P1*) of ca. 40 µV for the left ear (increased), and 10 µV for the right ear (normal). Scale y axis: 5 μV. **e** Normalization of the oVEMP n10p15 amplitude (*N1P1*) for the left ear post-surgery (3 μV), unchanged amplitude for the right ear (10 μV). Scale y axis: 2 μV

**1)** **≥** **1 symptom consistent with the presence of a “third mobile window” in the inner ear**
Bone conduction hyperacusis (*autophony, hearing one’s own internal body sounds in the affected ear, e.g., eye movements, bowel sounds, footsteps*)
Sound-induced vertigo and/or oscillopsia time-locked to the stimulus (*Tullio phenomenon*)
Pressure-induced vertigo and/or oscillopsia time-locked to the stimulus
Pulsatile tinnitus
**2)** **≥** **1 of the following signs or diagnostic tests indicating a “third mobile window” in the inner ear**
Nystagmus characteristic of excitation or inhibition of the affected superior semicircular canal, evoked by sound (*≥* *100* *dB nHL*) or by changes in middle ear or intracranial pressure (*e.g., Valsalva test, Hennebert sign, Politzer test*)
Low-frequency negative bone conduction thresholds on pure-tone audiometry (*typically for frequencies ≤* *1000* *Hz, often also increased air conduction thresholds in this frequency range* *→* *inner ear conductive hearing loss;* Fig. 2b)
Enhanced VEMP responses (low cVEMP thresholds or high oVEMP amplitudes; Fig. 2d)
**3) High-resolution temporal bone CT imaging with multiplanar reconstruction demonstrating dehiscence of the superior semicircular canal ***(recommended slice thickness ≤* *0.2* *mm; CBCT also possible; reconstruction in Pöschl plane (parallel to the superior semicircular canal) and Stenvers plane (perpendicular to it))*
**4) Not better accounted for by another vestibular disease or disorder**

Additions to the original text of the classification are shown in italics

*ICVD* International Classification of Vestibular Disorders, *c* cervical, *CT* computed tomography, *CBCT* cone beam CT, *nHL* normal hearing level, *o* ocular, *VEMP* vestibular evoked myogenic potential

In cases of suspected third window syndrome, always check for negative BC thresholds in the PTA

If this particular measurement is omitted, the result is a seemingly “normal” PTA (Fig. 2a), and the important differential diagnostic clue of negative BC thresholds (Fig. 2b) is lost. In this context, it should be noted that BC thresholds of the contralateral (healthy) ear may also be negative in unilateral SCDS due to difficulties in masking [40].

After surgical closure of the third window, normal biomechanics and fluid dynamics of the inner ear are restored, leading to a regression of clinical signs and symptoms and a normalization of the pathognomonic audiovestibular test findings (Fig. 2c, e; [7, 9, 17]).

## Superior canal dehiscence syndrome

Superior canal dehiscence syndrome is diagnosed according to the ICVD criteria (International Classification of Vestibular Disorders) from the synthesis of characteristic clinical symptoms/signs, pathognomonic electrophysiological findings, and the radiological evidence of a bony dehiscence of the superior semicircular canal (superior canal dehiscence, SCD) in high-resolution computed tomography (HRCT) or cone beam computed tomography (CBCT) of the temporal bone (Table 1; [40]). Since both the presence and the size of the dehiscence are often overestimated in imaging, further diagnostic tests with high specificity are necessary to identify those patients who actually suffer from a clinically relevant superior canal dehiscence *syndrome* (SCDS) and thus may benefit from surgical closure of the dehiscence [26].

### Vestibular evoked myogenic potentials

Vestibular evoked myogenic potentials have been used in the diagnosis of SCDS and other third window syndromes for over 20 years. Accordingly, there are numerous studies with different electrode localizations (cervical (c-) and ocular (o‑) VEMPs), stimulus qualities (air-conducted sound and bone-conducted vibration), stimulus parameters (stimulus frequency, morphology, repetition rate), and measurement parameters (VEMP amplitudes, latencies, thresholds) as well as different conclusions about the diagnostic accuracy of different cut-off values. A detailed compilation and a discussion of these results have been provided by Noij and Rauch [26].

Enhanced VEMP responses (increased amplitudes, reduced thresholds) are characteristic of SCDS

An example is shown in Fig. 2d, while the pathophysiological basis is described in [6] and [17]. In summary, the following VEMP findings are typically observed in SCDS (further details are presented in Table 2):

1. oVEMP amplitudes measured at 500 Hz air-conducted sound (ACS) provide better discrimination between SCDS and normally encased labyrinths than cVEMP thresholds for click stimuli—with shorter measurement time and lower noise exposure [18, 40, 45].
2. The presence of VEMP responses to high-frequency stimuli (e.g., cVEMPs at 2 kHz, oVEMPs at 4 kHz) is considered a strong indicator for the presence of SCDS. At these high frequencies, no VEMP responses are usually elicited in the intact bony labyrinth [5, 21, 25, 35].
3. If high-frequency VEMP recordings are not available, it is recommended to combine several measurement parameters at lower frequencies to improve diagnostic accuracy, e.g., using the “Third Window Indicator” (TWI = cVEMP threshold at 500 Hz ACS *minus* ABG at 250 Hz). At 2 kHz stimulus frequency, however, the TWI provides no further benefit in diagnostic accuracy compared to cVEMP thresholds alone ([25]; Table 2).
4. Cut-off values have been proposed for various VEMP parameters (Table 2). These depend on several factors (e.g., applied stimulus parameters, employed diagnostic criteria for SCDS, control population) and should be determined for different age groups in each vestibular laboratory [18, 45]. It is generally recommended to use stimuli with a short rise time in order to achieve the highest possible VEMP amplitudes [5].
5. Despite their high sensitivity and specificity, enhanced VEMP responses alone usually do *not* provide 100% diagnostic accuracy for the presence of an SCDS—especially when discriminating between SCDS and other vestibular syndromes (Table 2). Possible differential diagnoses for enhanced VEMP responses include, for example, other third window syndromes, intravestibular schwannomas, or Meniere’s disease in the irritable stage [7].

**Table 2:** Literature review of cut-off values of various oVEMP and cVEMP parameters in the diagnosis of superior semicircular canal dehiscence syndrome

Study	SCDS definition	Control group	Measurement and stimulus parameters	Cut-off value	Sensitivity (%)	Specificity (%)
*oVEMP amplitude (500* *Hz ACS)*						
Janky et al. (2013) [18]	Intraoperative confirmation	Age-matched HCs with no hearing or vestibular deficits	oVEMP n10 amplitude 4 ms stimulus (1/2/1)^1^ 125 dB SPL	> 8.25 µV	100	100
Zuniga et al. (2013) [45]	Intraoperative confirmation	Age-matched HCs with no hearing or vestibular deficits	oVEMP n10p15 amplitude 4 ms stimulus (1/2/1) 125 dB SPL	> 17.1 µV	100	98
Verrecchia et al. (2019) [37]	Symptoms + audio-vestibular tests + CT	Dizzy patients without SCDS in a tertiary neurotological center	oVEMP n10p15 amplitude 4 ms stimulus (1/2/1) 125 dB SPL	> 16.7 μV	100	89
*oVEMP amplitude (500* *Hz BCV)*						
Manzari et al. (2012) [22]	Clinical + CT	HCs without vestibular disorders	oVEMP n10 amplitude 7 ms stimulus (1/5/1) 130 dB FL at Fz^2^	> 10 μV	100	97
*oVEMP amplitude (4* *kHz ACS)*						
Manzari et al. (2013) [21]	Clinical + CT	HCs without vestibular disorders	oVEMP n10 amplitude 7 ms stimulus (1/5/1) 120 dB SPL	> 0 μV	100	100
Tran et al. (2020) [35]	Ward criteria (2017) [39]	Patients without SCDS in a tertiary neurotological center	oVEMP n10p15 amplitude 5 ms stimulus (2/1/2) 95 dB nHL	> 0 μV	86.5	87.7
Tran et al. (2020) [35]	Ward criteria (2017) [39]	Patients without SCDS in a tertiary neurotological center	oVEMP n10p15 amplitude 5 ms stimulus (2/1/2) 95 dB nHL	> 15 μV	83.8	96.8
*oVEMP amplitude (4* *kHz BCV)*						
Manzari et al. (2013) [21]	Clinical + CT	HCs without vestibular disorders	oVEMP n10 amplitude 7 ms stimulus (1/5/1) 130 dB FL at Fz^2^	> 0 μV	100	100
*cVEMP threshold (500* *Hz ACS)*						
Noij et al. (2018) [25]	Clinical + CT	HCs without hearing loss, vertigo, balance problems	cVEMP threshold “2 cycle rise and fall times” (4/0/4)	< 98 dB peSPL	52	100
Noij et al. (2018) [25]	Clinical + CT	HCs without hearing loss, vertigo, balance problems	500 Hz TWI (= cVEMP threshold at 500 Hz ACS—ABG at250 Hz)	< 103 dB	88	100
Tran et al. (2020) [35]	Ward criteria (2017) [39]	Patients without SCDS in a tertiary neurotological center	cVEMP threshold 5 ms stimulus (2/1/2)	< 75 dB nHL	55.6	96
*cVEMP threshold (2* *kHz ACS)*						
Noij et al. (2018) [25]	Clinical + CT	HCs without hearing loss, vertigo, balance problems	cVEMP threshold “2 cycle rise and fall times” (1/0/1)	< 118 dB peSPL	92	100
Noij et al. (2018) [25]	Clinical + CT	HCs without hearing loss, vertigo, balance problems	2 kHz TWI (= cVEMP threshold at 2 kHz ACS—ABG at 250 Hz)	< 92 dB	92	100

*ABG* air–bone gap, *ACS* air-conducted sound, *BCV* bone-conducted vibration, *Fz* midline of forehead at the hairline, *FL* force level, *HC* healthy control, *nHL* normal hearing level, *(pe)SPL* (peak equivalent) sound pressure level, *SCDS* superior canal dehiscence syndrome,* TWI* Third Window Indicator

^1^ (Rise time/plateau/fall time) of the stimulus, duration in ms

^2^ Fz: midline of the forehead at the hairline

Many of the features described above are also observed in other third window syndromes, but evidence from the literature is more scarce compared with SCDS.

Measurement of oVEMP amplitudes at 4 kHz is a fast and reliable “single-step test”

### Electrocochleography

Electrocochleography (ECochG) measures electrical potentials generated by acoustic stimulation of the cochlea via insert phones (clicks of 100 μs duration at 85 dB nHL [normal hearing level] or long tone bursts of 8 ms duration). Electrical activity of the cochlea is recorded via a promontorial needle electrode or a surface electrode on the eardrum. The signals are averaged 1000–1500 times analogous to brainstem audiometry. This test can be performed either under local or general anesthesia. Electrocochleography is an umbrella term for the recording of various signal components, such as auditory nerve neurophonics, cochlear microphonics, summation potentials (SP), and action potentials (AP). As early as in the 1980s, ECochG was suggested for the diagnosis of Meniere’s disease by William Gibson [13] and has been used ever since [11, 16, 23, 34].

As with Meniere’s disease, the SP signal (proportional to the deflection of the basilar membrane and stimulation of the nerve fibers; [43]) and the AP signal (potential of the cochlear nerve) are used for the diagnosis of SCDS. Latencies and amplitudes vary with stimulus strength [10]: Amplitudes increase proportionally with increasing acoustic stimulation, while latencies become shorter. The ECochG signals also vary with intracochlear pressure, which has been shown intraoperatively during a Valsalva maneuver [12]. Changes in the ratio between the SP and AP amplitudes indicate a fistula or a third window: A disproportion is to be expected in 76.5% [41] to 89% [1] of SCDS cases. The patients showed an average SP/AP ratio of 0.62 with a cut-off value of > 0.4. The specificity is 70% compared to HRCT as the diagnostic gold standard (not considering near-dehiscence syndromes [3] and the clinical presentation of the SCDS). The aforementioned cut-off values vary depending on the measurement set-up and must therefore be determined in healthy individuals by the respective user. One possible explanation for the enhanced SP/AP ratio in SCDS—analogous to endolymphatic hydrops in Meniere’s disease—is a reduction in perilymphatic pressure due to the bony dehiscence possibly resulting in a relative overpressure of the endolymph (*hydrops e vacuo*; [41]).

**Fig. 3:**
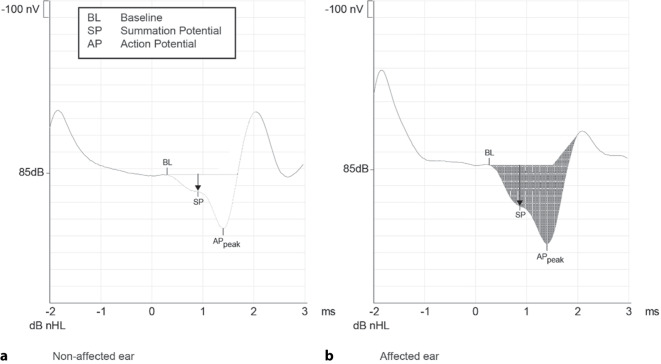
Electrocochleographic measurement of the amplitudes for summation potentials (*SP*) and action potentials (*AP*) with an air conduction stimulus of 85 dB nHL (normal hearing level). **a** Normal findings: SP/AP = 0.32. **b** Ear with superior canal dehiscence syndrome: SP/AP = 0.52

Figure 3a shows an ECochG recording of a healthy individual with normal amplitude ratios (SP/AP). The ratio between the summation potential and the action potential (0.32) is calculated from the amplitudes or the areas under the curve. In a patient with SCDS (Fig. 3b), a pathological SP/AP ratio > 0.4 (0.52) can be seen.

An SP/AP ratio > 0.4 in the ECochG supports the diagnosis of SCDS

In addition to diagnostics, ECochG can also be used for intraoperative monitoring of SCD surgery [1, 41], as the signal changes/normalizes after closure of the dehiscence (resurfacing and/or plugging; [2]).

However, there is still a lack of standardization in the application of this test. No intraoperative ECochG signal can be measured in over 20% of cases [29]. This may be due to various factors, such as electromagnetic interference, fluid in the ear canal, tilted insert phones, middle ear effusion, or electrode placement [29, 30]. There is also a large variance in the standard values, which is why this test has not yet become established in everyday clinical practice. This variance is not only dependent on the placement and type of electrodes, but also on the selected stimulus, the band filter (e.g., 3.2 Hz to 3 kHz), and the patient’s inner ear function [11]. As ECochG is semi-invasive depending on the type of electrode used, many specialists are hesitant to use it; therefore it has not yet been included in the current diagnostic criteria of the Bárány Society [40]. Nonsurgical ENT specialists and neurologists often prefer noninvasive methods such as c‑/o-VEMPs as additional electrophysiological tests for the diagnosis of a fistula or a third window syndrome. Nevertheless, ECochG is an important additional examination for confirming a third window syndrome in view of the many possible differential diagnoses.

### Wideband tympanometry

In wideband tympanometry, an ACS stimulus with a frequency spectrum of 226–8000 Hz is applied to the eardrum, and the sound absorption by the middle/inner ear is determined for the individual frequencies. In accordance with the underlying pathophysiology of SCDS (i.e., reduced impedance of the inner ear for frequencies < 1000 Hz), increased sound absorption was measured for SCDS patients in this frequency range. Furthermore, the resonance frequency is shifted to lower frequencies. After successful surgical closure of the dehiscence, these findings normalize [36]. As only data from a small number of patients have been published so far, the sensitivity and specificity of this method for the diagnosis of SCDS or other third window syndromes cannot yet be assessed. Further studies are needed to determine its additional benefit in everyday clinical practice [9].

### Video head impulse test

In recent years, isolated hypofunction (= reduced gain + corrective saccades) of the affected superior semicircular canal in the vHIT has been reported in patients with SCDS. This finding is certainly useful in differentiating SCDS from other diagnoses. Possible underlying pathomechanisms for gain reduction include (incomplete) auto-plugging of the affected semicircular canal by the overlying dura or shunting of mechanical energy during quick head turns through the SCD (“endolymphatic flow dissipation”; [4]).

### Vibration-induced nystagmus

Despite being a simple, quick, and sensitive test for the diagnosis of SCDS, VIN is still rarely used in clinical practice. In summary, this “vestibular Weber test” utilizes the increased sensitivity of the inner ear for bone conduction stimuli in SCDS. When a BCV stimulus with a frequency between 60 and 800 Hz is applied to the vertex, 88% of SCDS patients display a stimulus-coupled nystagmus beating in the direction of the affected ear in 95% of cases and a maximum response at approx. 400 Hz. According to Ewald’s first law, stimulation of the superior semicircular canal results in a predominantly vertical-torsional nystagmus. An overview of the methodology and application of VIN in SCDS can be found in [8], and the neurophysiological principles are explained in [6].

## Syndrome of the large vestibular aqueduct

**Fig. 4:**
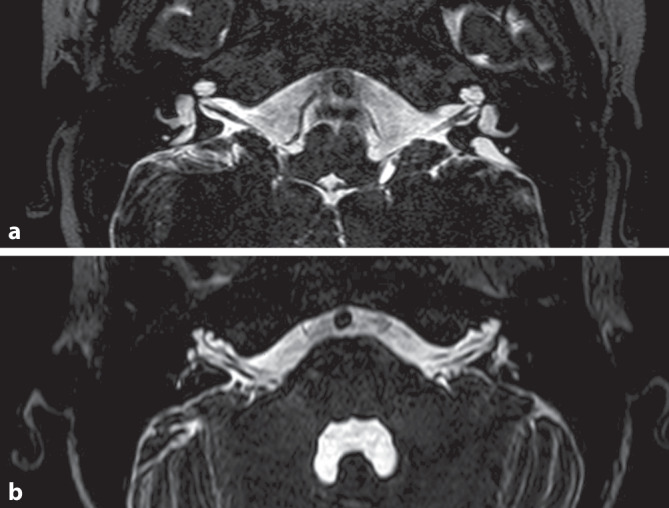
T2-weighted magnetic resonance imaging of the cerebellopontine angle in two patients (**a,** **b**). Axial plane. (From [27] © S. Rösch et al., CC BY 4.0, https://creativecommons.org/licenses/by/4.0/). **a** Patient with a clearly enlarged vestibular aqueduct on both sides, dorsal to the horizontal semicircular canal. **b** Patient with bilateral X‑linked malformation of the cochlea with a bilateral missing modiolus (“incomplete partition type III”)

An enlarged vestibular aqueduct (EVA) is an anatomical variant in the area of the dorsal temporal bone, which can be recognized in various imaging techniques due to its irregular enlargement ([32]; Figs. 1 and 4a). According to the Cincinnati criteria, an EVA is defined as follows on axial HRCT of the temporal bone: diameter of the bony vestibular aqueduct > 1.9 mm (operculum) or > 0.9 mm (midpoint between vestibule and operculum) [38].

EVA is one of the most common malformations of the petrous temporal bone

The presence of an EVA in combination with characteristic clinical symptoms is also referred to as large vestibular aqueduct syndrome (LVAS). The atypical morphology of the bony canal (vestibular aqueduct) and the membranous endolymphatic duct located therein is considered to be caused by disorders of pH and volume regulation during embryogenesis [14], resulting in changes of the surrounding structures. An EVA is described as one of the most common malformations of the petrous temporal bone [42], but cannot be assigned to a specific clinical picture. Rather, it represents a clinical indicator for a possible genetic cause of a clinical phenotype [27].

Variants in the *SLC26A4* gene are the most common genetic causes and are significantly more frequently detected in EVA patients of Asian origin than in European (Caucasian) patients [27]. Other genes whose variants have been repeatedly described in association with EVA in Caucasian patient cohorts are *FOXI1, KCNJ10, POU3F4, GJB2, TMC1* [27], and *CHD7* [28], as well as a haploid genotype (haplotype) upstream of the *SLC26A4* gene [15]. The plethora of possible causative genes leads to a variety of different clinical manifestations. There have been descriptions of EVA in “non-syndromic hearing loss with EVA” (DFNB4) as well as in syndromic disorders such as Pendred syndrome, distal renal tubular acidosis with hearing loss, CHARGE syndrome, Waardenburg syndrome, branchio-oto-renal syndrome, and branchio-oculo-facial syndrome. Therefore, genetic testing should be considered in the presence of an EVA for clarification of the underlying cause, targeted counseling, and early detection of possible further pathologies [19].

Always consider a thyroid assessment (Pendred syndrome) and genetic testing in cases of EVA

In analogy with the different underlying causes, heterogeneous clinical phenotypes are described for EVA, both with regard to audiological and vestibular findings [15, 20, 44]. Hearing impairment often occurs pre- or perilingually and is usually progressive. Several aspects may contribute to the type of hearing loss being diagnosed (conductive, sensorineural or combined): In addition to the time point of the hearing examination (e.g., early and mild form of hearing impairment with a possibly still visible conductive component), the applied audiometric procedures are also decisive for categorization of the hearing impairment, especially in young children. Measurement of bone conduction thresholds is crucial for the detection of a possible ABG as part of a third window phenomenon. Finally, an EVA may only be recognized during preoperative imaging before cochlear implantation for profound sensorineural hearing loss. In these cases, preexisting clinical symptoms can only be aligned to a third window syndrome retrospectively.

Vestibular disorders are less frequently described in EVA patients. Clinical reports also vary widely in this regard. Both VEMP measurements [44] and vHIT examination results [20] are often abnormal in EVA patients. The incongruent results between caloric and vHIT testing are similar to those of patients with endolymphatic hydrops (Meniere’s disease; [33]).

## X-chromosomal malformation of the cochlea

This malformation (Fig. 4b), also known as “incomplete partition type III” [31], which is radiologically conspicuous owing to the absence of the modiolus, represents a further anatomical variant that can be the cause of a third window phenomenon due to the abnormal widening of the internal auditory canal and the incomplete bony separation between the cochlea and the internal auditory canal. The X‑linked malformation is often associated with mutations of the *POUF3F4* gene, affects predominantly male patients, and is associated with an ipsilateral EVA in about 50% of cases [27]. Due to the association with a perilymph gusher upon opening the otic capsule, the syndrome is also called “X-linked hearing loss with stapes gusher” (*DFNX2*; [7]).

The audiological and vestibular symptoms are similar to those of EVA. The frequently observed accompanying fixation of the stapes footplate, which may also contribute to a conductive hearing loss, may complicate the interpretation of the audiological findings and the resulting treatment plan.

## Practical conclusion

- The diagnosis of a third window syndrome always results from the synthesis of symptoms, clinical signs, pathognomonic audiovestibular tests, and imaging.
- When performing high-resolution computed tomography/cone beam computed tomography of the temporal bone, ensure that the slice thickness is sufficiently thin (ideally ≤ 0.2 mm) and that the reconstruction is multiplanar (Pöschl and Stenvers planes for superior canal dehiscence syndrome).
- Always evaluate the computed tomography scans yourself—for the presence of superior canal dehiscence and beyond!
- If an enlarged vestibular aqueduct is present, consider other inner ear malformations and a possible involvement of other organs (e.g., Pendred syndrome!).

## Abbreviations

ABG: Air–bone gap
ACS: Air-conducted sound
AP: Action potential
BC(V): Bone-conducted (vibration)
c: Cervical
CBCT: Cone beam computed tomography
ECochG: Electrocochleography
EVA: Enlarged vestibular aqueduct
FL: Force level
HRCT: High-resolution computed tomography
ICVD: International Classification of Vestibular Disorders
LVAS: Large vestibular aqueduct syndrome
nHL: Normal hearing level
o: Ocular
pe: Peak equivalent
PTA: Pure-tone audiometry
SCD: Superior canal dehiscence
SCDS: Superior canal dehiscence syndrome
SP: Summating potential
SPL: Sound pressure level
TWI: Third window indicator
VEMPs: Vestibular evoked myogenic potentials
vHIT: Video head impulse test
VIN: Vibration-induced nystagmus

## Declarations

For this article no studies with human participants or animals were performed by any of the authors. All studies mentioned were in accordance with the ethical standards indicated in each case.

The supplement containing this article is not sponsored by industry.
